# Change in patient concerns following total knee arthroplasty described with the International Classification of Functioning, Disability and Health: a repeated measures design

**DOI:** 10.1186/1477-7525-6-112

**Published:** 2008-12-11

**Authors:** Ravi Rastogi, Bert M Chesworth, Aileen M Davis

**Affiliations:** 1 Physiotherapist, London Health Sciences Centre, London, Ontario, Canada; 2 Assistant Professor, School of Physical Therapy, Faculty of Health Sciences and Department of Epidemiology and Biostatistics, Schulich School of Medicine and Dentistry, The University of Western Ontario, London, Ontario, Canada; 3 Senior Scientist, Health Care and Outcomes Research Division and Arthritis Community Research Evaluation Unit, Toronto Western Research Institute; Associate Professor, University of Toronto, Toronto, Ontario, Canada

## Abstract

**Background:**

There is no published evidence of how patient concerns change during the first six weeks following total knee arthroplasty (TKA). An understanding of the recovery process from the patient's perspective will inform clinicians on how to best educate patients about their post-operative concerns. Our objectives were to (1) quantify the level of importance for each of 32 previously identified concerns pre-operatively, and across the first six weeks following primary TKA and, (2) convey this change in importance post-operatively using the components of the International Classification of Functioning, Disability and Health (ICF).

**Methods:**

The objectives were achieved using a repeated measures design. Convenience sampling was used to recruit 54 consecutive patients undergoing primary TKA at a hospital in Ontario, Canada. Pre-operatively and at two, four and six weeks post-operatively subjects rated the level of importance for each of the 32 previously identified patient concerns

**Results:**

The importance rating of patient concerns in all four ICF components changed from before surgery to two weeks after surgery. Patient concerns in the Participation component became increasingly important after the first two weeks following surgery. Post-operatively from week two to week four, changes in importance ratings were also found in the Body Function and Activity components, but not in the Environmental Factors component.

**Conclusion:**

Changes in patient concerns mirror their early recovery from TKA surgery. Consistent with this, Participation restrictions become increasingly important to patients after discharge from acute care suggesting that clinicians should think of managing patient expectations for return to societal roles early in post-operative rehabilitation.

## Background

Osteoarthritis (OA) of the knee is a common cause of pain and disability in the elderly population [1]. Depending on the severity of the disability, total knee arthroplasty (TKA) has been shown to be an effective procedure in reducing pain, improving function and quality of life in individuals suffering from OA of the knee [2,3]. According to the Canadian Joint Replacement Registry [4], the number of TKA procedures in Canada increased by more than 100% from 1994/95 to 2004/05 with two-thirds of all knee replacements in the 65–84 year age group. As the baby boomer population approaches this age group, it is expected that this trend will continue.

When evaluating the success of any treatment, opinions of patients are of great significance [5]. This is especially important for elective procedures, such as TKA, which are normally performed to improve the individual's quality of life [6]. Different researchers have demonstrated that patient expectations play an important role in determining the outcome following TKA [7]. To the extent that patient expectations and concerns may be related, it is essential that health care providers appropriately address concerns that are important to patients to maximize outcomes following TKA.

Even though patients are routinely referred for physical therapy in the acute post-operative phase (0–6 weeks) after TKA, there is no published evidence of how patient concerns change during this early period of recovery. An understanding of the recovery process from the patient's perspective will inform the clinician's approach to patient education during rehabilitation.

Many of the outcome measures used to quantify change following primary TKA are based on patients' perceptions of their status, but the choice of outcomes is rarely grounded in a conceptual framework [8,9]. The International Classification of Functioning, Disability and Health (ICF) released by the World Health Organization [10]) provides a unified framework for evaluating health and health-related states of populations and also provides a common language for communication among health care professionals [11,12]. The ICF model includes two parts, with each part containing separate components. The first part covers functioning and disability and includes the components of Body Structure and Function, Activities, and Participation. The second part covers contextual factors and includes the components of Environmental Factors and Personal Factors. The World Health Organization [10] has indicated that it is difficult to differentiate between Activities and Participation as defined by the ICF. Others believe that it is necessary to differentiate between these components if the ICF is to be widely accepted to describe functioning in rehabilitation [13,14].

In an earlier study, to identify concerns that patients had about their recovery from surgery, we performed individual face-to-face cross-sectional interviews with 30 patients during the first six weeks following primary TKA for OA. All patient concerns were identified by asking patients to answer one specific question, "What is important to you right now with respect to your recovery from knee replacement surgery?". During the interview it was emphasized that patients should only identify what was important during that particular week of the interview. Patients were allowed to report as many concerns as they wished, all of which were recorded verbatim in writing. The primary investigator (RR) conducted all interviews. We grouped patient comments on the basis of common themes, identifying 32 concerns about surgical recovery that we then linked to the components of the ICF [15]. Seven of these concerns were linked to the Body Function component of the ICF while 15, 4 and 4 concerns were linked to the Activity, Participation and Environmental Factors components, respectively. Receiving appropriate information regarding what to expect with rehabilitation following surgery and being independent were the only two concerns that could not be linked to the ICF. The primary objectives of this study were to (1) quantify the level of importance for each of these concerns pre-operatively and across the first six weeks following primary TKA and, (2) convey this change in importance post-operatively using the components of the ICF.

## Methods

Participants were English-speaking ambulatory patients with knee OA who were waiting for a primary TKA. Sample size calculations were based on a concurrent study of responsiveness of the WOMAC [unpublished]. Convenience sampling was used to recruit consecutive patients from the waiting lists of orthopaedic surgeons working in a large tertiary care hospital. Each subject participated in four evaluation sessions: pre-operatively and at two, four and six weeks after surgery. During each evaluation session subjects rated the level of importance for each of the 32 concerns identified in our earlier study [15] and were also given the opportunity to provide additional concerns. Importance was measured on a seven-point scale (1 = not important at all, 2 = important to a very small extent, 3 = important to a small extent, 4 = important to a moderate extent, 5 = important to a fairly great extent, 6 = important to a great extent, 7 = important to a very great extent). At each evaluation session, subjects also completed the Numeric Pain Rating Scale (NPRS) [16] and the Knee Injury and Osteoarthritis Outcome Score (KOOS) [17] to measure knee-related pain intensity and patient perceived health status, respectively. The Western Ontario McMaster Universities Osteoarthritis Index (WOMAC) is embedded within the KOOS. Therefore, scores for the subscales of the WOMAC were extracted from the KOOS. Both the KOOS and the WOMAC have good psychometric properties and are commonly used to assess patient perceived health status among TKA recipients [17,18]. Demographic and clinical characteristics including comorbidities using the Self-Administered Comorbidity Questionnaire [19] were also captured pre-operatively. The University of Western Ontario Research Ethics Board for Health Sciences Research Involving Human Subjects approved this study.

### Analysis

Descriptive statistics were used to summarize the pre-operative characteristics of subjects. We described change in clinical status between each time point by evaluating each subscale of the KOOS with a repeated-measures analysis of variance (ANOVA) and Bonferroni post-hoc testing [20]. To quantify how the level of importance changed for patient concerns during the first six weeks after TKA, the appropriate measures of central tendency and dispersion were calculated for each concern at each one of the four evaluation sessions. To describe change in the importance ratings using the ICF components we did the following for each subject at each time point of data collection. We assigned a single level of importance to each ICF component by using the median importance rating from the concerns within that ICF component. To evaluate the change in this value across time, for each ICF component we conducted a Friedman two-way ANOVA by ranks [21]. The null hypothesis was that there would be no difference in the mean rank of importance across time [21]. When a significant difference was found, post-hoc testing between each time point was performed with the Wilcoxon signed-ranks test [20]. Because four separate nonparametric ANOVAs were performed and we could potentially perform 12 post-hoc tests between time points, we applied the Bonferroni correction factor [20] and considered p = 0.003 (e.g. 0.05/16) as the threshold for significance.

## Results

**Figure 1:**
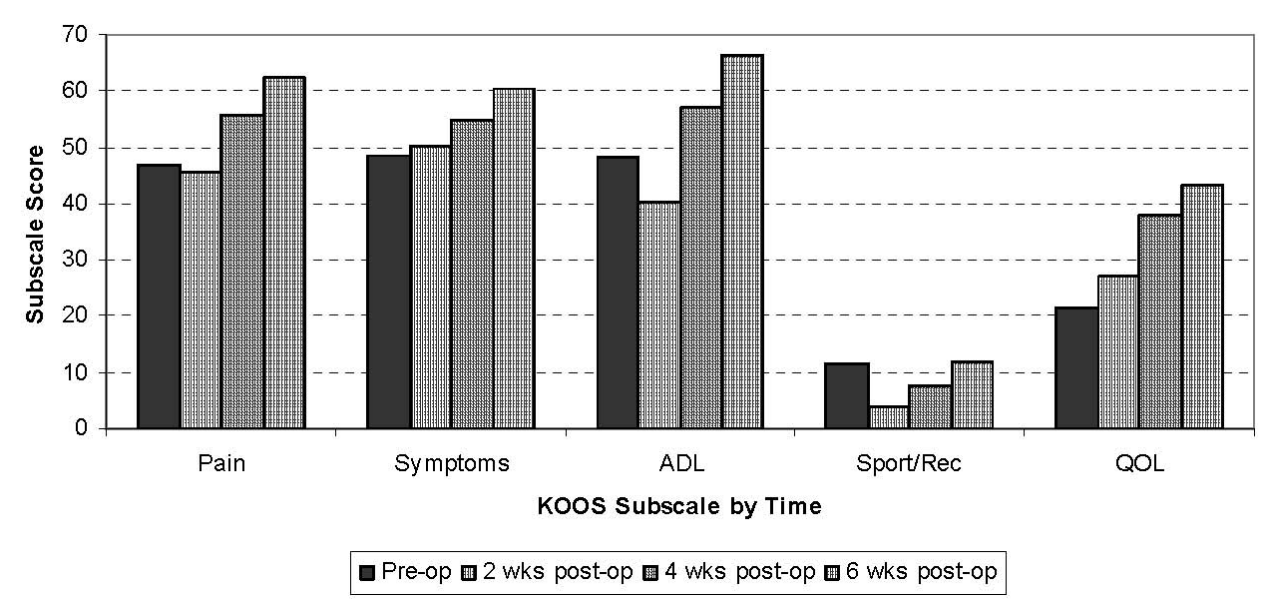
Average KOOS* score grouped by subscale: pre-operatively (pre-op) and two, four and six weeks after (post-op) primary total knee arthroplasty (n = 54). * KOOS = Knee Injury and Osteoarthritis Outcome score: subscales are Pain, other Symptoms, function in daily living (ADL), function in sport and recreation (Sport/Rec) and knee-related Quality of life (QOL). Subscale scores can vary from 0–100 with 100 indicating the best state.

Fifty-seven subjects were contacted and consented to take part in the study. One person withdrew from the study without any explanation and two people did not return phone calls to set up an appointment, leaving 95% of recruits who completed the study (n = 54). There were no missing data. Of the 54 subjects who completed the study, 48% (n = 26) were men. The average age (standard deviation) of the sample was 68.1 (8.9) years. Clinical characteristics of the subjects along with their preoperative scores for the KOOS, WOMAC and NPRS are shown in Table 1. Values for the KOOS Function in Daily Living (ADL) subscale and the WOMAC Function subscale are identical because the items in the two subscales are the same. Figure 1 shows the mean scores by time for each KOOS subscale. The KOOS scores can range from 0–100 with 100 indicating best state. The ANOVA results indicated significant change over time for all subscales (p < 0.0001). Post hoc testing confirmed significant changes in the Pain and Knee-related Quality of Life (QOL) subscales only beyond two weeks following surgery (p = 0.01 to p < 0.0001). The ADL and Function in Sport and Recreation (Sport/Rec) subscales changed significantly in each 2-week period (p = 0.01 to p < 0.0001). Significant change in the Symptoms subscale was found between four and six weeks after surgery (p = 0.02).

**Table 1:** Pre-operative subject characteristics (n = 54).

Walking aid, *n(%)*	
None	32 (59)
Cane	15 (28)
Four wheeled walker	7 (13)
	
Number of comorbidities, median(Q_1_-Q_3_)*min- max^†^	3 (2–4) 1–6
	
Five most prevalent comorbidities, *n(%)*	
High Blood Pressure	32 (59)
Back Pain	24 (44)
Diabetes	15 (28)
Heart Disease	12 (22)
Depression	10 (19)
	
KOOS subscale scores§ *mean (SD^‡^)*	
Pain	46.9 (15.1)
Symptoms	48.4 (14.9)
ADL	48.3 (16.9)
Sport/Rec	11.5 (14.6)
QOL	21.6 (14.5)
	
WOMAC subscale scores§ *mean (SD)*	
Pain	51.5 (16.4)
Stiffness	41.4 (16.6)
Function	48.3 (16.9)
	
Numeric Pain Rating Scale^||^, *mean (SD), min-max*	6.4 (2.1) 1–10

* (Q1-Q3) = 25^th ^to 75^th ^percentile

^† ^min-max = minimum to maximum

^‡ ^SD = Standard deviation

^§ ^KOOS = Knee Injury and Osteoarthritis Outcome score: subscales are Pain, other Symptoms, function in daily living (ADL), function in sport and recreation (Sport/Rec) and knee-related Quality of life (QOL). WOMAC = Western Ontario and McMaster Universities Osteoarthritis Index: subscales are Pain, Stiffness and Function. Subscale scores can vary from 0–100 with 100 indicating the best state

^|| ^Values on the scale can vary from 0 = no pain to 10 = worst possible pain

As mentioned earlier, during each evaluation session subjects were given the opportunity to identify any additional concerns regarding their recovery from knee replacement surgery. No additional concerns were provided by subjects at any of the four evaluation sessions. On visual inspection, the importance ratings for the 32 concerns identified in our earlier study [15] were skewed. Therefore the median and first and third quartiles were used to summarize the distribution of these ratings. Tables 2, 3, 4 show these values across time for each concern within an ICF component. They show that before surgery, most concerns had relatively high importance ratings, with some remaining fairly stable after surgery and others changing more dramatically over time. Median levels of importance for the two concerns that were not covered by the ICF (receiving appropriate information regarding what to expect with rehabilitation following your surgery and being independent) had high levels of importance at all four time points (i.e. either 6 or 7 on the 7-point scale).

**Table 2:** Importance levels for patient concerns linked to the ICF* Body Function component: pre-operatively (pre-op) and two, four and six weeks after knee arthroplasty (n = 54)

**Patient Concern**	**Pre-op**	**Week 2**	**Week 4**	**Week 6**
Decreasing pain in your surgical knee	6 (5–7) ^†^	7 (6–7)	6 (5–7)	6 (4–6)
Reducing the swelling in your surgical leg	3 (1–6)	6 (5–7)	6 (5–7)	5 (3–6)
Avoiding infection in your surgical knee	2 (1–7)	7 (6–7)	5 (2–7)	3 (1–6)
Sleeping better at night	5 (4–7)	6 (5–7)	6 (5–7)	6 (4–7)
Increasing the bend in your surgical knee	4 (2–6)	7 (6–7)	6 (5–7)	6 (5–7)
Increasing the straightening in your surgical knee	4 (1–5)	6 (5–7)	5 (4–6)	6 (3–6)
Increasing the strength in your legs	6 (4–7)	6 (5–7)	6 (5–7)	6 (5–6)

* International Classification of Functioning, Disability, and Health

^† ^median (25^th ^to 75^th ^percentile); importance ratings are on a 7-point scale (1 = not important, 7 = important to a very great extent)

**Table 3:** Importance levels for patient concerns linked to the ICF* Activity component: pre-operatively (pre-op) and two, four and six weeks after knee arthroplasty (n = 54)

**Patient Concern**	**Pre-op**	**Week 2**	**Week 4**	**Week 6**
Getting out of bed on your own	4.5 (3–7)	6 (5–7)	5 (3–6)	5 (3–7)
Getting in/out of bath	5 (3–7)	1 (1–4)	5 (3–6)	4 (2–6)
Putting on your own shoes or socks	5 (3–6)	6 (4–7)	5 (4–6)	5 (4–6)
Dressing yourself	4 (2–6)	5.5 (4–7)	5.5 (4–6)	6 (4–7)
Walking on a flat surface	5 (4–7)	6 (5–7)	6 (4–7)	6 (4–7)
Walking on uneven ground	5 (3–7)	1 (1–3)	3 (1–5)	4 (3–6)
Descending stairs	6 (4–7)	3.5 (1–6)	5 (3–6)	5 (4–6)
Ascending stairs	6 (5–7)	4 (1–6)	5 (3–6)	5 (4–6)
Cooking your own meals	5 (2–7)	1 (1–3)	4 (2–6)	4 (2–6)
Doing your own housework	5 (3–6)	1 (1–3)	3 (2–6)	4 (2–6)
Heavy domestic duties	2.5 (1–6)	1 (1–1)	1 (1–2)	1 (1–3)
Light domestic duties	4 (3–6)	2 (1–3)	4 (3–5)	4.5 (3–6)
Getting in/out of car	6 (4–7)	4 (2–6)	6 (4–7)	6 (5–7)
Comfortably sit in car	5 (3–6)	4 (2–6)	6 (4–6)	5.5 (4–6)
Doing your exercises as prescribed by your PT	4 (1–7)	7 (6–7)	6 (6–7)	6 (5–7)

* International Classification of Functioning, Disability, and Health

^† ^median (25^th ^to 75^th ^percentile); importance ratings are on a 7-point scale (1 = not important, 7 = important to a very great extent)

**Table 4:** Importance levels for patient concerns linked to ICF* Participation and Environmental Factors components: pre-operatively (pre-op) and two, four and six weeks after knee arthroplasty (n = 54)

**Patient Concern by ICF component**	**Pre-op**	**Week 2**	**Week 4**	**Week 6**
**Participation**				
Driving a vehicle	6 (3–7)	1 (1–1)	1 (1–4)	3.5 (1–6)
Going shopping	5 (3–6)	1 (1–1)	3 (1–5)	4 (3–6)
Returning to your hobbies	5 (2–7)	1 (1–2)	2 (1–3)	3 (1–5)
Going back to your regular exercise class or sport	4 (1–7)	1 (1–1)	1 (1–4)	3 (1–6)

**Environmental factors**				
Being less of a burden on your spouse or caregiver	6 (4–7)	6 (5–7)	6 (5–7)	6 (5–7)
Having the support of your family members	6 (5–7)	6 (6–7)	6 (5–7)	6 (5–7)
Having the support of your neighbours	3 (1–5)	3 (1–5)	4 (1–5)	4 (1–6)
Receiving competent care from health care workers in a timely manner	2.5 (1–6)	7 (6–7)	6 (5–7)	6 (4–7)

* International Classification of Functioning, Disability, and Health

^† ^median (25^th ^to 75^th ^percentile); importance ratings are on a 7-point scale (1 = not important, 7 = important to a very great extent)

**Figure 2:**
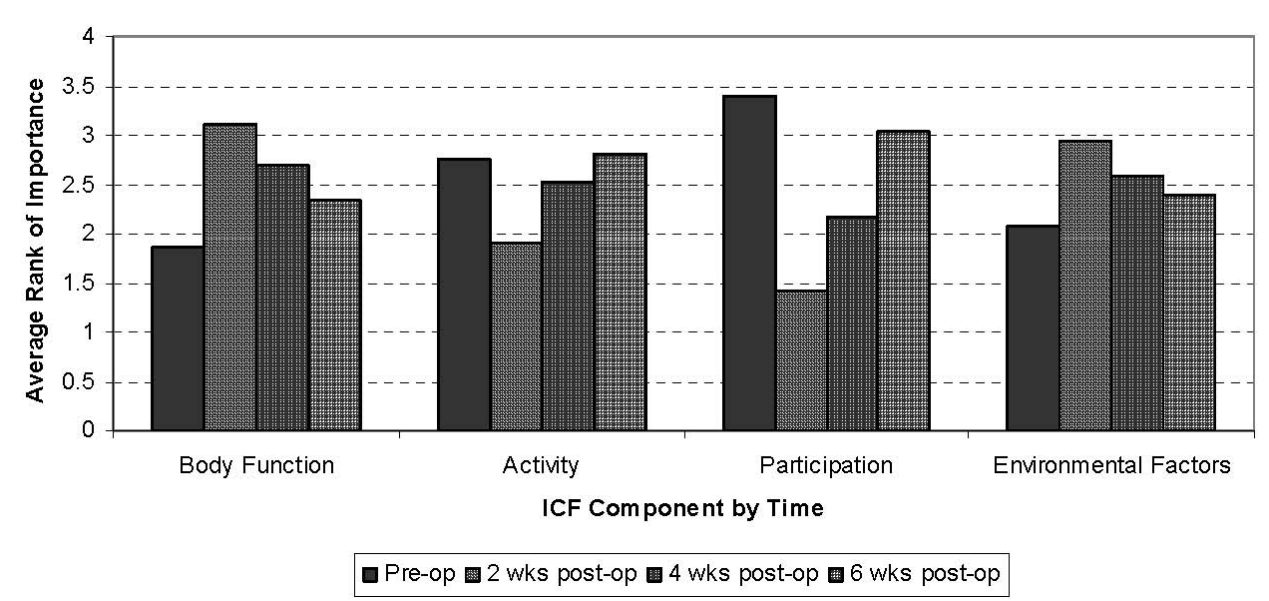
Average rank of importance for patient concerns grouped by ICF* component: pre-operatively (pre-op) and two, four and six weeks after (post-op) primary total knee arthroplasty (n = 54). * International Classification of Functioning, Disability, and Health

The Friedman's ANOVA results are shown in Figure 2. The mean rank of the importance rating changed over time (p < 0.0001) for all four ICF components (Body Function: χ^2^_3df _= 34.29; Activity: χ^2^_3df _= 20.61; Participation: χ^2^_3df _= 90.91; Environmental Factors: χ^2^_3df _= 14.37). Post-hoc testing revealed a significant change in importance between the pre-operative evaluation and post-operative week two for all four ICF components (p < 0.0001). Participation was the only ICF component that demonstrated a significant change in importance between all four-time points (p < 0.0001). The importance of the Body Function and Activity components changed significantly from before surgery through to post-operative week four (p < 0.001).

## Discussion

This study was conducted to gain a quantitative understanding of how the importance of patient concerns change during the first six weeks following primary TKA. It showed that the importance of some concerns change over time while others do not. In the discussion that follows we suggest these changes reflect the clinical aspects of the delivery of care and the early recovery process. We also propose that the importance ratings provide direct evidence that supports the differentiation of the Activity and Participation components of the ICF in the clinical context of this study. To the best of our knowledge, this is the first study of this nature.

The Friedman's ANOVA revealed the importance rankings of all four ICF components changed over time. Post hoc testing showed that concerns in the ICF Body Function component were ranked higher at post-operative week two compared to before surgery. The WHO has defined Body Function [10] as physiological functions of the body systems including psychological functions. Following major joint surgery such as TKA, physiological functions perceived as important by patients, such as motion of the knee joint, strength in the lower extremities and sleeping at night are commonly impaired for the first month post-operatively [6]. The sensation of pain and swelling in the surgical leg are also common occurrences during this phase of post-operative recovery. Presence of these impairments after surgery would naturally cause discomfort and limit normal daily activities. Consistent with this logic, patients in this study rated these Body Function concerns as being more important to them in the first month following surgery.

Going through surgery and recovery may also explain the pattern of change in the Activity and Participation importance ratings. Post hoc testing showed that patients ranked concerns in these components as being less important two weeks after surgery. Due to the nature of the surgery it is common for patients to experience impairments with certain body functions (increased pain and swelling, decreased knee range of motion, impaired sleep), which generally lead to limitations in the activities that they can perform. Researchers have reported a decline in physical function in the first month following TKA surgery [6]. Consistent with this, patients in our study demonstrated significant decreases in the KOOS ADL and Sport/Rec subscales in the first two weeks after surgery. Due to this decline, concerns from the ICF Activity component (e.g. 'walking on uneven ground', 'cooking your own meals', 'doing your own housework') and concerns from the Participation component (e.g. 'driving a vehicle', 'shopping' or 'returning to hobbies') dropped in importance during the first two weeks post-operatively. Their subsequent increase in importance to pre-operative levels after surgery, may reflect the fact that patients are generally starting to feel better, are mobilizing with greater ease and are becoming less dependent on their caregivers. Indeed, Kennedy and colleagues [22] and Stratford and Kennedy [23] found that physical function improved to pre-operative levels by post-operative week six to eight. Again, consistent with this, patients in our study showed significant increases in the KOOS ADL and Sport/Rec subscales from two weeks through to six weeks post-operatively. The importance ratings for Body Function, Activity and Participation, combined with parallel changes in these KOOS subscale scores therefore, supports the validity of these concerns and their importance to patients because they mirror the surgical and early rehabilitation focus.

Just like the Body Function component, patient concerns that mapped to the Environmental Factors component were ranked higher two weeks after surgery. Due to the immediate decline in physical function [6], patients require the support of family members and health care workers to help them manage with daily activities and restore their physical function. Consistent with this, concerns such as 'having the support of your family members', 'receiving competent care from health care workers' and 'being less of a burden on your spouse or caregiver' were important to patients during this acute post-operative phase of their recovery. 'Having the support of your neighbours' was the only concern mapping to the Environmental Factors component that was of moderate to small importance at each post-operative evaluation session. This may relate to the fact that TKA is an elective surgery, which allows the patient time to plan for their post-operative needs using alternative resources (home care, family members etc.) and therefore, not requiring the support of their neighbours.

Two concerns that were not covered by the ICF components, 'receiving appropriate information regarding what to expect with rehabilitation following your surgery' and 'being independent' were of great importance to patients before surgery and remained at the same level of importance throughout the first six weeks of recovery. The first emphasizes the importance of education from the patient perspective. In this regard, Soever and MacKay [24] have documented that receiving information about their rehabilitation is important to patients and that this type of education improves patient satisfaction following total joint replacement surgery. The second highlights the consistently high value that patients place on independence. Hinojosa and Youngstrom have reported that "independence is defined by the individual's culture and values, support systems, and ability to direct his or her life" [25]. Gignac and Cott [26] have reported that a loss of independence may have consequences on the quality of life and psychosocial well being of an individual. The cultural background of patients in this study and the support system in our society along with the consequences of losing independence, may explain why patients consistently rated the importance of this concern so high, throughout the first six weeks of recovery.

There is considerable debate regarding the need to distinguish between the Activity component and the Participation component of the ICF [12-14,27]. While the WHO decided not to distinguish between these two components; others have stressed the importance of their differentiation if the ICF is to be widely used when describing health and health-related states of various populations across the world [12-14]. The results of this study highlight the challenge one faces in differentiating the ICF Activity and Participation components. On one hand, the average ranking of importance across time for these two components (Figure 2) reveals a similar pattern over time, suggesting there is little to be gained from separation of the ICF components when describing the importance of patient concerns about recovery. On the other hand, the relatively low values in the KOOS Sport/Rec subscale (Figure 1) may explain the pattern in Participation concerns after the two-week mark following surgery. Only those concerns in the ICF Participation component showed consistent increases in their level of importance through the first six weeks after surgery. We believe this highlights one tangible benefit from separating the two components in the context of this work: patients are starting to think about their return to participation in roles early in the recovery phase. As evidenced by the KOOS Sport/Rec subscale, they are also aware of their limitations in Participation-related activities. This is important for the clinician to note as researchers have shown that patient expectations are a predictor of functional outcome and satisfaction following total joint replacement surgery [28]. Therefore, patient education should encompass return to participation roles very soon after surgery and in particular, should re-evaluate participation-related goals from two to six weeks of postoperative care. Future studies should determine if the increasing importance assigned to Participation concerns in this study continues to increase beyond the six-week mark post-operatively.

All participants in the study were recruited from a single tertiary care hospital. This may be viewed as decreasing the generalizability of this study to other settings. Even though the results may not be applicable to all TKA populations, the patient demographics and pre-operative functional status findings were comparable to that of other TKA studies [17,29-31]. The rehabilitation setting (e.g. home care, in-patient or out-patient facilities) following surgery may influence what is important to patients. The majority of patients in this study received their initial therapy at home after acute care discharge until approximately three weeks following surgery. Then they continued therapy at an out-patient clinic of their choice. Therefore, it is possible that patients undergoing TKA, who receive therapy in a different setting than described above may have different priorities and concerns during the first six weeks following surgery. We note however that no subjects in this study provided additional concerns when given the opportunity to do so at four different time points of data collection. Furthermore, when the importance findings are viewed in combination with the KOOS data, changes in patient concerns mirrored the early recovery pattern from TKA surgery. This supports the validity of the concerns we investigated and combined with the finding that importance levels varied over time, suggests a temporal element that should be included in future work of this nature. As there was no test-retest component in this study, reliability of the importance ratings at each time point of data collection could not be confirmed. Another limitation of this study was the inability to include non-English speaking individuals. Therefore, the results are not applicable to non-English speaking recipients of TKA surgery. Finally, our sample size for this study was based on sample size calculations of a concurrent study of responsiveness of the WOMAC [unpublished]. This seemed acceptable because we had no previous importance ratings on which to base our sample size calculations. Where no significant differences were found, it may be possible the study was underpowered.

## Conclusion

In conclusion the study shows the level of importance that patients assign to concerns about recovery from TKA surgery, is a reflection of the stage they are in, during the surgical and rehabilitation process. In particular, Participation restrictions become increasingly important to patients from two to six weeks after surgery, suggesting that patients do think about their return to activities at the societal level early in their postoperative rehabilitation. Clinicians can use this knowledge to integrate their approach to education, goal setting and managing expectations about return to participation roles after TKA surgery.

## Competing interests

The authors declare that they have no competing interests.

## Authors' contributions

RR, AMD and BMC designed the study. RR collected and analyzed the data and drafted the manuscript with regular feedback from AMD and BMC. All authors read and approved the final manuscript.
